# Co-Immobilization of Lactase and Glucose Isomerase on the Novel g-C_3_N_4_/CF Composite Carrier for Lactulose Production

**DOI:** 10.3390/nano12234290

**Published:** 2022-12-02

**Authors:** Le Wang, Bingyu Jiao, Yan Shen, Rong Du, Qipeng Yuan, Jinshui Wang

**Affiliations:** 1 National Engineering Laboratory for Wheat & Corn Further Processing, College of Biological Engineering, Henan University of Technology, Zhengzhou 450001, China; 2 State Key Laboratory of Chemical Resource Engineering, Beijing University of Chemical Technology, Beijing 100029, China

**Keywords:** g-C_3_N_4_/CF, carbon fiber, co-immobilization, lactulose, enzyme stability

## Abstract

The g-C_3_N_4_/CF composite carrier was prepared by ultrasound-assisted maceration and high-temperature calcination. The enzyme immobilization using the g-C_3_N_4_/CF as the novel carrier to immobilize lactase and glucose isomerase was enhanced for lactulose production. The carbon fiber (CF) was mixed with melamine powder in the mass ratio of 1:8. The g-C_3_N_4_/CF composite carrier was obtained by calcination at 550 °C for 3 h. After the analysis of characteristics, the g-C_3_N_4_/CF was successfully composited with the carbon nitride and CF, displaying the improvement of co-immobilization efficiency with the positive effects on the stability of the enzyme. The immobilization efficiency of the co-immobilized enzyme was 37% by the novel carrier of g-C_3_N_4_/CF, with the enzyme activity of 13.89 U g^−1^ at 60 °C. The relative activities of co-immobilized enzymes maintained much more steadily at the wider pH and higher temperature than those of the free dual enzymes, respectively. In the multi-batches of lactulose production, the relative conversion rates in enzymes co-immobilized by the composite carrier were higher than that of the free enzymes during the first four batches, as well as maintaining about a 90% relative conversation rate after the sixth batch. This study provides a novel method for the application of g-C_3_N_4_/CF in the field of immobilizing enzymes for the production of lactulose.

## 1. Introduction

Compared with traditional catalysts, biological enzymes have attracted more and more attention due to their high catalytic activity, high substrate specificity, and mild catalytic conditions, and have been widely used in biotechnology and industrial fields [1].

However, the poor stability of free enzymes and the difficulty of recovery bring some challenges to large-scale industrial production. The fragile nature of free enzymes and the narrow operating temperature and pH range limit their commercial use [2]. Immobilization has been reported to be an effective strategy for improving enzymes’ stability and achieving low-cost recovery [3]. The choice of carrier material has a great influence on the catalytic effect of immobilized enzymes [4,5]. Adsorption is the fastest and most common method for immobilizing enzymes [6], and is used to immobilize laccase, resulting in improved preservation and pH stability, which maintained about 40% relative viability (4 °C, 30 d) and more than 50% relative viability in the pH range of 2.0−6.0 [7]. The co-immobilized dual-enzyme biocatalyst was successfully prepared by immobilizing horseradish peroxidase (HRP) and glucose oxidase (GOD) in a covalently bound manner on dopamine (DA)-modified cellulose (Ce)-chitosan (Cs) composite spheres, with the degradation rate of the co-immobilized dual enzyme biocatalyst remaining at 61.2% after 6 batches of degradation [8]. The old yellow enzyme (OYE3) was immobilized by acetal-agarose covalent binding (OYE3-GA) and affinity adsorption of EziGTM particles (OYE3-EziG). In the bioreduction reaction of α-methyl-trans-cinnamaldehyde, the OYE3-GA could be recycled for up to 12 reaction cycles, with a maximum conversion of 40% after 12 cycles [9]. However, immobilized enzymes also had some drawbacks [10]; for instance, the enzymatic activity and catalytic reaction rate of the immobilized enzymes were lower than the free enzymes. This is maybe due to the fact that the immobilization process changes the natural spatial structure of the enzymes’ molecules [11] and reduces the freedom of movement of the immobilized enzymes’ molecules, thus reducing the effective collision between the enzymes and the substrate. Moreover, the microenvironments around the enzymes’ molecules change [12]. When multiple enzymes are immobilized, their active sites may be affected by various factors [13], such as the selection of the carrier [14,15], the immobilization method of the enzyme [6], changes in the microenvironment around the enzyme molecule [16], the reaction conditions [17], and others. In addition, the bioactivity of proteins may be affected by the stability of primary and higher structures, including the secondary, tertiary, and quaternary structures. Therefore, in the field of immobilized enzymes, various methods had been devised to preserve the conformation of proteins and thus avoid loss of activity. As the key determinant of immobilization technology, the selection of the immobilized carrier was very important.

Carbon fiber (CF) has become a common immobilized carrier material because of its high physical and chemical stability, good biocompatibility, and abundant active functional groups. Currently, CF is used for the immobilization of various enzymes, such as lipase [18], laccase [19], glucose oxidase, and so on [20]. However, the untreated CF had a smooth surface, inertia, and weak adsorption with enzymes. In order to further expand the application of CF in the immobilization field, researchers often modify it or prepare composite carriers. The application of composite carriers in immobilization technology has also been reported, such as CF/graphene [21], CF/carbon nanotubes, etc. [22].

Graphite phase carbon nitride (g-C_3_N_4_) is the most stable allotrope of carbon nitride, with excellent thermal and chemical stability [23]. In addition, the raw materials used in the preparation of g-C_3_N_4_, such as melamine, dicyandiamide, melamine, urea, etc., are inexpensive [10]. Therefore, a g-C_3_N_4_/CF composite carrier had the highest practical application value. The amino group at the edge of g-C_3_N_4_ can bind to enzymes by physical or covalent bonding to form a more stable complex [1]. In addition, g-C_3_N_4_ has high biocompatibility and a surface-active center, making it an ideal material for the immobilization of enzymes [24]. Glucose isomerase was immobilized on g-C_3_N_4_ nanosheets to prepare a novel sensitive biosensor for glucose detection under neutral conditions [25]. Glucose isomerase immobilized on a glass-carbon electrode was modified with an Au-g-C_3_N_4_ nanocomposite (Gox/Au-g-C_3_N_4_), establishing a sensing platform in the presence of luminal to detect glucose in samples [26].

g-C_3_N_4_/CF composite carriers have attracted extensive attention from researchers, but are currently mainly used in the field of photocatalysis [23,27,28], while there are few reports in the field of immobilization. It is noteworthy that the introduction of g-C_3_N_4_ could greatly improve the surface roughness, functional groups, and wettability of CF [29], which is conducive to the immobilization of enzymes.

Two or more enzymes were fixed on the same carrier to produce a cascade reaction that facilitates product separation and simplifies catalyst reuse [30]. In addition, due to the co-immobilization of different catalysts on the same carrier, the diffusion limitation of intermediates between active sites was reduced [31], which improves the overall reaction efficiency in the one-pot method [32]. Compared with the single immobilized enzyme, the overall performance of the immobilized double enzymes was improved [33]. The co-immobilization of enzymes could reduce mass transfer resistance and improve enzyme activity through effective substrate channels, and enhance reusability and stability [33]. The cellulose and glucose oxidase were co-immobilized on GO by covalent bonding, which showed that the co-immobilized double enzymes retained about 65% of their initial activity after seven repetitions [34].

Lactulose is a disaccharide synthesized from galactose and fructose, which does not exist in nature [35], and which has received increasing attention for its significant health-promoting effects [36]. Lactulose can be broken down and utilized by beneficial bacteria in the colon, and is widely added to processed foods as a probiotic [37]. It can also be used in the pharmaceutical industry for the treatment of hepatic encephalopathy and constipation [38]. As a high-value product, lactulose has many physiological functions, such as effectively promoting the proliferation of intestinal bacteria, inhibiting the growth of potential pathogenic bacteria, increasing the production of beneficial metabolites, and enhancing the absorption of minerals by the intestinal tract [39]. Lactulose has a wide range of applications in the food, pharmaceutical, and health food industries [40].

At present, the synthesis of lactulose mainly includes chemical and biological methods. Electrolysis is an effective technique to produce lactose from whey lactose [41]. The application of electroactivation (EA) technology for isomerization of lactose to lactofructose in an EA reactor is conditioned by anion and cation exchange membranes [42]. The glutaraldehyde-activated chitosan was used as a carrier to immobilize lactase, which resulted in an efficient and stable catalyst for the synthesis of lactofructose using cheese whey and fructose as substrates [43]. Lactulose was synthesized from fructose and lactose in continuous packed-bed reactor operation with glyoxyl-agarose immobilized *Aspergillus oryzae* β-galactosidase [44]. Lactulose synthesis with biological enzymes could overcome some disadvantages of industrial chemical synthesis, such as the degradation of lactulose, side reaction, purification of late products, and so on [45]. Lactose can be converted into galactose and glucose under the catalysis of lactase [46]. Glucose was isomerized to fructose by glucose isomerase, then fructose and galactose were linked by a β-1, 4 glycosidic bond to synthesize lactulose under the catalysis of lactase [47,48]. Glucose isomerase isomerized glucose to fructose, which reduced its inhibition on lactose hydrolysis and accelerated the process of lactose hydrolysis-glucose isomerization [49]. However, using lactose as a single substrate, the synthesis of lactulose by lactase and glucose isomerase will lead to a low lactulose yield [50]. In the initial stage of the reaction, the fructose substrate was insufficient. In the later stage, when the fructose content accumulated to the transglycoside reaction suitable for lactase, the lactose content decreased greatly and the transglycoside reaction was difficult to continue, resulting in a low lactulose content. Consequently, the appropriate amount of fructose could be added at the initial stage of the reaction to increase lactulose yield.

**Figure 1:**
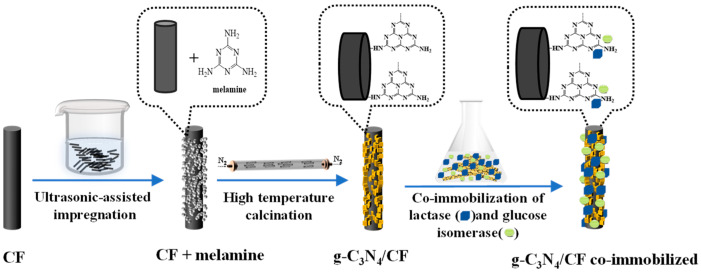
Preparation process diagram of the g-C_3_N_4_/CF composite carriers for co-immobilization. The acetone-treated CF was ultrasonically impregnated in melamine saturated solution for 1 h, dried, then their surfaces were covered with melamine powder uniformly according to different ratios. After that, they were calcined at 550 °C for 3 h under the nitrogen protection and cooled to produce a g-C_3_N_4_/CF composite carrier. The g-C_3_N_4_/CF composite carrier was combined with enzyme molecules and finally the co-immobilization of enzymes with the g-C_3_N_4_/CF as the novel composite carrier was obtained.

In our research, the novel carriers, using g-C_3_N_4_/CF and different amounts of melamine and CF, were prepared for the co-immobilization of lactose and isomeric glucose, as shown in Figure 1. The characterizations of composite carriers were performed to investigate the physicochemical properties of g-C_3_N_4_/CF for co-immobilization. Moreover, the effects of g-C_3_N_4_/CF on the co-immobilization of lactase and glucose isomerase were elucidated for the lactulose production.

## 2. Materials and Methods

### 2.1. Chemicals and Reagents

CF was purchased from Shanghai Yingjie Special Fiber Materials Co., Ltd. (Shanghai, China). Melamine (AR) was purchased from Tianjin Dingsheng Xin Chemical Co., Ltd. (Tianjin, China). Acetone (AR) and Coomassie Brilliant Blue (AR) were purchased from Sinopharm Group Chemical Reagent Co., Ltd., (Shanghai, China). Lactase was purchased from Harbin Meihua Biotechnology Co., Ltd., (Harbin, China). Glucose isomerase was purchased from Genenko Bioengineering Co., Ltd., (Wuxi, China). Bovine serum albumin (AR) was purchased from Shanghai Zhanyun Chemical Co., Ltd. (Shanghai, China). Lufluorescein isothiocyanate (FITC, ≥95%) was purchased from Biosharp Biotechnology Co., Ltd. (Hefei, China). O-nitrophenol (99%) was purchased from Shanghai Maclin Biochemical Technology Co., Ltd., (Shanghai, China). O-Nitrophenyl-β-D-galactopyranoside (ONPG AR) purchased from Shanghai Baoman Biotechnology Co., Ltd. (Shanghai, China). Fuctose (AR), lactose (AR), and rhodamine B, were purchased from Tianjin Comio Chemical Reagent Co., Ltd. (Tianjin, China). Lactulose (99%) was purchased from Sigma (Shanghai, China).

### 2.2. Preparation of g-C_3_N_4_/CF Composite Carriers

The g-C_3_N_4_/CF composite carriers were prepared by ultrasonic-assisted impregnation and high temperature calcination. The CF was impregnated in acetone solution for 5 h then dried at 50 °C to obtain the pretreated CF (all CF mentioned below refers to the pretreated CF). During the preparation of the typical g-C_3_N_4_/CF composite carrier, the CF was added to saturated melamine aqueous solution by ultrasonic impregnation for 1 h and melamine powder was coated evenly on the surface of the wet CF (m (CF): m (melamine) = 1:2, 1:4, 1:6, 1:8, 1:10) and this was placed in a crucible with the initial dried in an oven at 50 °C. The crucible was covered with a lid and completely wrapped in aluminum foil. The crucible was calcined in N_2_ at 550 °C for 3 h, and the heating rate was 5 °C·min^−1^. After cooling at room temperature, the prepared g-C_3_N_4_/CF composite carrier was washed in water to remove the loose g-C_3_N_4_/CF on the surface of the CF, then dried and reserved (the prepared samples were named g-C_3_N_4_/CF-1, g-C_3_N_4_/CF-2, g-C_3_N_4_/CF-3, g-C_3_N_4_/CF-4, and g-C_3_N_4_/CF-5).

In addition, the pure g-C_3_N_4_ was synthesized by calcining melamine directly in N_2_ at 550 °C for 3 h at a heating rate of 5 °C min^−1^.

### 2.3. Co-Immobilization and SDS-PAGE Analysis of Lactase and Glucose Isomerase

Lactase (2.5 g, 5.9 U g^−1^) and glucose isomerase (1 g, 58.9 U g^−1^), with an activity ratio of 1:4, were dissolved in 50 mL of 0.2 mol L^−1^ PBS solution at pH 6.0 and 0.5 g of immobilized carrier (Raw-CF, g-C_3_N_4_/CF-1, g-C_3_N_4_/CF-2, g-C_3_N_4_/CF-3, g-C_3_N_4_/CF-4, g-C_3_N_4_/CF-5) was added. The reaction was vibrated at 30 °C at 160 r min^−1^ for 5 h. The immobilized enzymes were obtained by filtration and washed 3 times with the PBS solution. Polyacrylamide gel electrophoresis (SDS-PAGE) was used to evaluate the protein profiles of the enzyme preparations used in this study.

### 2.4. Selection of the Composite Carrier with Enzyme Activity Ratio and the Kinetics of Co-Immobilized Enzymes

The optimal immobilized carrier was determined by the enzyme activity recovery and co-immobilization efficiency. Lactase and glucose isomerase were immobilized in the best immobilization carrier according to the activity ratios of 1:0, 1:1, 1:3, 1:4, 1:5, and 1:6 to obtain the best enzyme activity ratio.

The Kms of the immobilized enzymes were determined according to Micellis–Menton kinetics with different concentrations of lactose/fructose solutions (containing 10% lactose and 2% fructose) as substrates.

### 2.5. Enzyme Activity Assays

Control assays were performed to determine the effect of the immobilization experimental conditions on the stability of each enzyme. A solution of 1% lactase and 1% glucose isomerase was prepared and the enzyme activities of each enzyme before and after the immobilization experimental conditions (30 °C, 160 r min^−1^, 5 h) were determined.

The enzyme activity recovery rate was calculated according to the following equation (Equation (1)).

With the bovine serum albumin as the standard protein, the amount of enzyme protein supported on an immobilized carrier was measured by using the Coomassie brilliant blue Bradford method by subtracting the residual protein content in the solution from the initial protein content [2]. The co-immobilization efficiency was calculated by Equation (2).

(1) $$\mathrm{The}\;\mathrm{enzymes}’\;\mathrm{activity}\;\mathrm{recovery}\;\mathrm{rate}\;(\%)=\frac{A_{i}}{A_{0}}\times 100$$

where A_0_ is the total enzymes activity of free enzymes, A_i_ is the total enzyme activity of the immobilized enzymes.

(2) $$\mathrm{Co}-\mathrm{immobilization}\;\mathrm{efficiency}\;(\%)=\frac{m_{0}-m_{i}}{m_{0}}\times 100$$

where m_0_ is the total protein content of initial enzyme solution (mg), m_i_ is the total protein content of residual liquid after immobilization (mg).

Lactose can be hydrolyzed into galactose and glucose by lactase [51], the hydrolysis process is expressed in Equation (3). O-Nitrophenyl β-D-galactopyranoside (ONPG) is a chromogenic substrate of lactase, suitable for the detection of lactase activity. Its color development principle is that lactase can hydrolyze ONPG to produce galactose and alkaline conditions of the yellow product o-nitrophenol. O-nitrophenol detected absorbance at 420 nm [52]. The lactase activity was measured using ONPG as substrate and a pH of 4.5 at 45 °C for 30 min. The absorbance was measured at 420 nm and the amount of o-nitrophenol released was determined. Under the above conditions, the enzyme activity unit was defined as the amount of enzyme required to produce 1 μmoL of o-nitrophenol per minute of ONPG consumption.

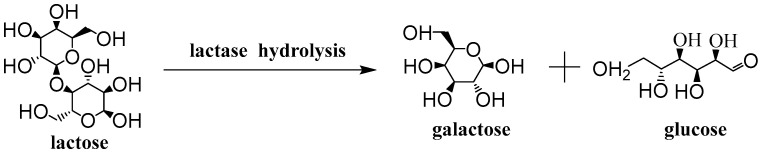
(3)

Glucose could be isomerized by glucose isomerase [49], the isomerization process is represented by Equation (4). The activity of glucose isomerase was measured using glucose as a substrate and a pH of 7.5 at 70 °C for 30 min. The fructose content was determined by HPLC. The unit of activity of glucose isomerase was defined as the amount of enzyme required to consume glucose per minute to produce 1 μmoL of fructose at pH 7.5, 70 °C.

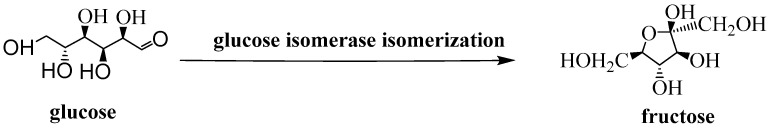
(4)

Galactose and fructose can be converted into lactulose by lactase [47]; the synthesis process of lactulose can be expressed by Equation (5). The free dual enzyme and co-immobilized enzyme activities were determined using a lactose/fructose solution (containing 10% lactose and 2% fructose) as a substrate, pH 6.0, at 55 °C for 4 h.

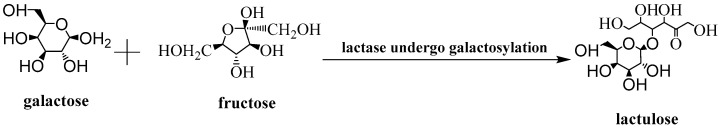
(5)

The concentrations of fructose and lactulose were determined by HPLC using a Hitachi HPLC system (Tokyo, Japan) and the N2000 software (Ejer Technol. Co., Ltd., Hangzhou, China). The specific method of HPLC was as follows. Column: sugar-pakTM1, 6.5 × 309 mm (Milford, MA, USA). Mobile phase: ultra-pure water. Flow rate: 0.5 mL min^−1^. Detector: refractive index detector (RID). Column temperature: 50 °C. Injection volume: 10 µL.

### 2.6. Characterization of Structure and Properties of Immobilized Enzyme with the g-C_3_N_4_/CF Composite Carriers

The carrier surface morphology and immobilized enzyme morphology were detected by scanning electron microscopy (SEM, Quanta 250FEG, FEI, Hillsboro, OR, USA). Lactase and glucose isomerase were labeled with rhodamine B and FITC, respectively, and immobilized on g-C_3_N_4_/CF composite carrier. The distribution of these two enzymes on the surface of g-C_3_N_4_/CF composite carrier was observed by a fluorescence microscope (Revolve FL, Echo Laboratories, San Diego, CA, USA). The specific surface area and pore size distribution of immobilized carrier were analyzed by the pore size and specific surface area tester (SSA, Biaode Electronic Technology Co., Ltd., Beijing, China). Fourier transform infrared spectroscopy (FTIR, WQF-530, Beijing Beifenruili Analytical Instrument Factory, Beijing, China) was used to detect the surface functional groups of the carrier. X-ray photoelectron spectroscopy (XPS, Nexsa, ThermoFisher, MA, USA) was used to detect the surface elemental composition and chemical states of the carrier. The chemical structure of the carrier was determined by X-ray diffractometer (XRD, Miniflex 600, Nippon Science Company, Tokyo, Japan)

### 2.7. Assay of Co-Immobilized Enzyme Activity

#### 2.7.1. Effect of Temperature and pH on Enzymes’ Activities

The enzymes and substrates reacted at 45−75 °C, and the enzymes’ activities were determined. The enzyme activity was the relative activity, which was set as the highest enzyme activity of 100%, to detect the optimal temperature of the enzymes. The free double enzymes and co-immobilized enzymes were incubated in PBS buffer at 65, 70, and 75 °C for different times, respectively, and the enzyme activities were determined.

The enzymes and substrates reacted at pH 4.5−7.5 and the enzyme activities were determined. The enzyme activity was the relative activity, which was set as the highest enzyme activity of 100%, to detect the optimal pH of the enzymes. The free double enzymes and co-immobilized enzymes were incubated in PBS buffer at pH 4.5, 6.0, and 7.5 for different times, respectively, and the enzyme activities were determined.

The enzyme activity was the relative activity, which was set as the highest enzyme activity of 100% to detect the thermal stability and pH stability of the enzymes, respectively. Enzyme assays were according to Section 2.6.

#### 2.7.2. Storage Stability of Co-Immobilized Enzymes

The free enzyme and co-immobilized enzyme were stored in PBS buffer with pH 6.0 at 4 °C for 30 days. The residual enzyme activity was measured every 5 days. The enzyme activity was the relative activity, which was set as the enzyme activity before storage of 100%. Enzyme assays were according to Section 2.6.

#### 2.7.3. Operational Stability of Co-Immobilized Enzymes

Co-immobilized enzymes and free double enzymes were respectively added to the lactose/fructose (containing 10% lactose and 2% fructose) substrate solution and reacted for 4 h at 65 °C. After the reaction, the lactulose content was determined, and the co-immobilized enzyme was recovered.

The co-immobilized enzyme continued to the fresh substrate solution and circulated for six times. The concentration of lactulose generated from the free enzyme in the first reaction was set to 100%, and the lactulose content generated by the co-immobilized enzyme relative to the free dual enzyme was recorded as the relative conversion rate of the co-immobilized enzyme. Enzyme assays were according to Section 2.6.

The relative conversion of co-immobilized to pectose was calculated by Equation (6).

(6) $$\mathrm{The}\;\mathrm{lactofructose}\;\mathrm{yield}\;\mathrm{rate}\;(\%)=\frac{C_{i}}{C_{0}}\times 100$$

where C_i_ was the content of lactulose produced by the co-immobilized enzyme, C_0_ was the content of lactulose produced by the free enzyme.

## 3. Results and Discussion

### 3.1. Structural Performance Characterization of the g-C_3_N_4_/CF Composite Carrier

**Figure 2:**
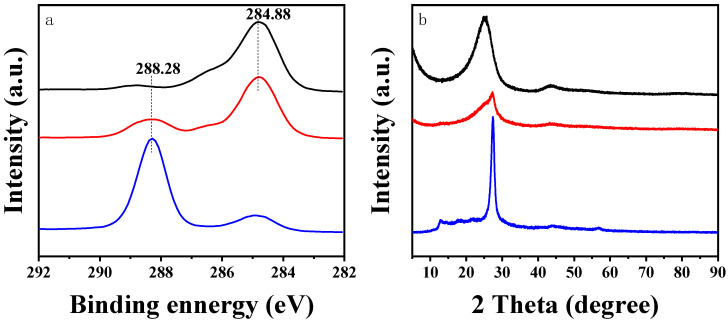
The XPS C1s spectra and the XRD profiles of the CF, g-C_3_N_4_, and g-C_3_N_4_/CF (CF: black line, g-C_3_N_4_/CF: red line, g-C_3_N_4_: blue line). (**a**)The XPS C1s spectra of CF, g-C_3_N_4_, and g-C_3_N_4_/CF. (**b**) The XRD profiles of CF, g-C_3_N_4_, and g-C_3_N_4_/CF.

The XPS C1s spectra and the XRD profiles of the CF, g-C_3_N_4_, and g-C_3_N_4_/CF are shown in Figure 2. The elemental compositions and chemical states of the surfaces of CF, g-C_3_N_4_, and g-C_3_N_4_/CF were determined by XPS. As shown in Table S1, the elements C, N, and O were present on the surfaces of CF, g-C_3_N_4_, and g-C_3_N_4_/CF. The introduction of g-C_3_N_4_ added more N elements to the surface of g-C_3_N_4_/CF compared to CF (10.31-fold increase).

In addition, XPS C1s spectra are shown in Figure 2a, to further understand the chemical states of CF, g-C_3_N_4_, and g-C_3_N_4_/CF surface elements. The CF had a characteristic peak at 284.88 eV which belonged to the sp^2^ hybrid C atom in the C–C group [53]. Compared with the CF, the characteristic peak located at 288.28 eV was increased in the XPS C1s spectrum of g-C_3_N_4_/CF, reflecting the sp^2^ bond C in the N=C(–N)_2_ group [29], which was caused by the introduction of g-C_3_N_4_. The above results indicate the composite with g-C_3_N_4_ on the CF surface.

The phases and structures of CF, g-C_3_N_4_, and g-C_3_N_4_/CF were determined by XRD. As shown in Figure 2b, the CF had only a wide diffraction peak at about 22°, which may be caused by the (002) plane of the graphite structure [27]. There were two characteristic diffraction peaks in g-C_3_N_4_, located at 13.1° and 27.4°, respectively. The peak at 13.1° was related to the in-plane ordered (100) plane of the trihomogeneous thiazine ring with a distance of 0.670 nm. The peak at 27.4° was related to the (002) plane of interlayer stacking reflection of conjugated aromatic system with an interlayer distance of 0.323 nm [28,29]. The diffraction peaks of the g-C_3_N_4_/CF composite carrier were similar to those of CF and g-C_3_N_4_, which were located at 22° (from CF), 13.1,° and 27.4° (from g-C_3_N_4_), respectively, indicating that the surface of CF was composited with g-C_3_N_4_.

Specific surface area and porosity have great influence on the immobilization effect of the carrier. The N_2_ adsorption–desorption isotherms and pore size distributions of different carriers as shown in Figure S1. Adsorption–desorption isotherms and pore size distributions of CF and g-C_3_N_4_/CF were measured by the BET method. The isotherms of CF and g-C_3_N_4_/CF were consistent with typical type II isotherms with hysteresis rings and shown in Figure S1a, which indicates that there is a complex pore structure on the surface of carriers [21]. As shown in Figure S1b, the aperture of the g-C_3_N_4_/CF composite carrier was mainly around 0.82 nm and 2.86 nm, while the aperture of CF was mainly between 2.4 and 5.74 nm. The results showed that, compared with CF, the pore structure of the g-C_3_N_4_/CF composite carrier was mainly microporous. Therefore, the adsorption capacity of g-C_3_N_4_/CF composite carriers was significantly higher than that of CF. The g-C_3_N_4_/CF composite carriers adsorption immobilization of biosynthetic enzymes was associated with physical interactions, including van der Waals forces, electrostatic interactions, hydrogen bonding, and ionic interactions [1].

The specific surface area, pore volume, and pore size of CF and g-C_3_N_4_/CF are shown in Table 1. The specific surface area of g-C_3_N_4_/CF was 5.08 m^2^ g^−1^, which was 8.33 times than that of CF. In addition, the microporous volume, mesoporous volume, and total pore volume of g-C_3_N_4_/CF were increased. The g-C_3_N_4_/CF composite carrier achieved a larger specific surface area and pore volume [23]. Within a certain range, the larger specific surface area and the smaller pore size may be favorable for the carrier adsorption of enzymes [25].

The above results indicate that compared with CF, a g-C_3_N_4_/CF composite carrier could achieve the adsorption immobilization of double enzymes by the virtue of excellent physical properties (such as rough surface structure, smaller pore size, larger specific surface area, and pore volume). Figure 2 indicates that the CF surface was loaded with g-C_3_N_4_. The g-C_3_N_4_ was the stable isomer in carbon nitride, with good biocompatibility, thermal stability, chemical inertia, electronic structure and so on. It is an ideal candidate for the preparation of immobilized enzymes [54,55].

### 3.2. The Apparent Morphology of the Co-Immobilized Enzyme and Its Distribution on the g-C_3_N_4_/CF

**Figure 3:**
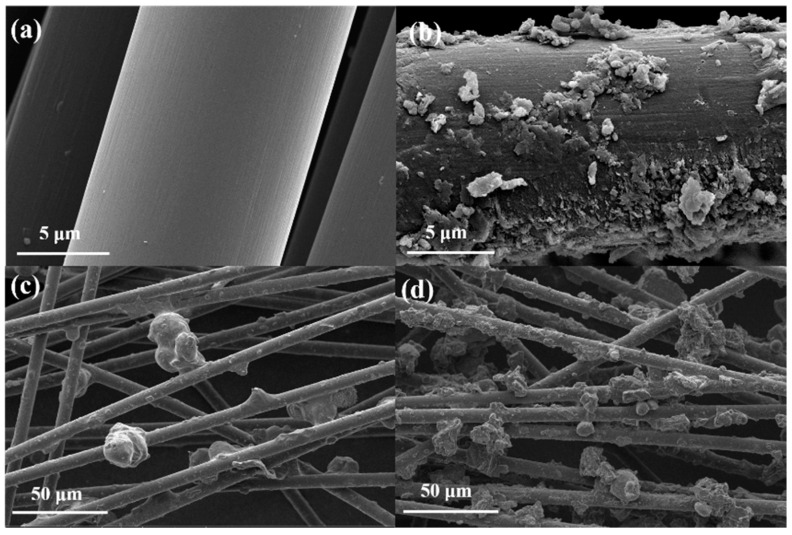
The SEM images of CF, g-C_3_N_4_/CF, and their immobilized enzymes. (**a**) CF; (**b**) g-C_3_N_4_/CF; (**c**) co-immobilized enzymes on CF; (**d**) co-immobilized enzymes on g-C_3_N_4_/CF.

**Figure 4:**
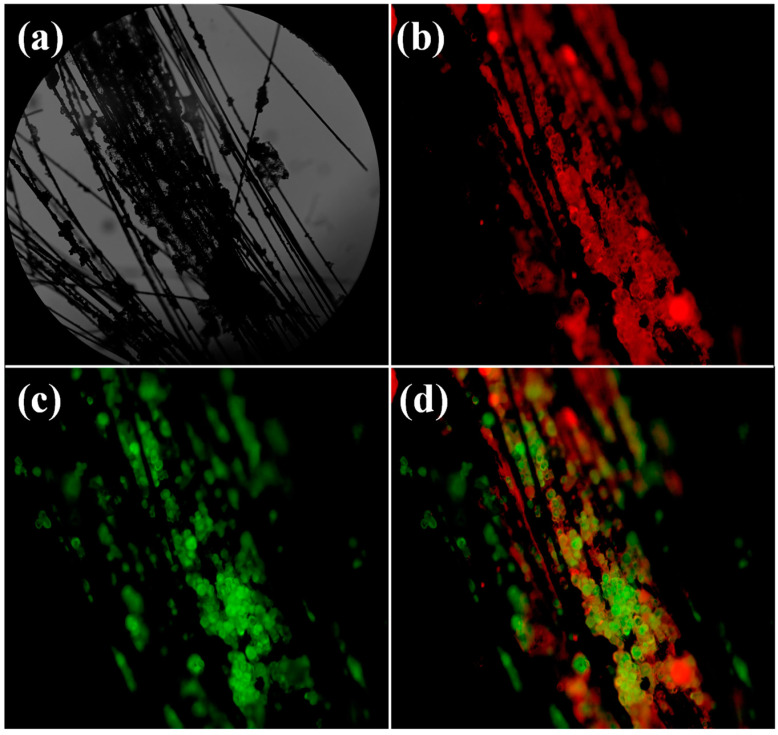
Distribution of double enzymes in g-C_3_N_4_/CF composite carrier. (**a**) Bright field image; (**b**) distribution of Rhoda mine B labeled lactase on g-C_3_N_4_/CF composite carrier; (**c**) distribution of FITC-labeled glucose isomerase on g-C_3_N_4_/CF composite carrier; (**d**) the distribution of lactase and glucose isomerase on g-C_3_N_4_/CF composite carrier. Herein, the g-C_3_N_4_/CF referred to the g-C_3_N_4_/CF composite carriers synthesized by being calcined with melamine and CF in 550 °C for 3 h.

The surface morphologies of CF, g-C_3_N_4_/CF, immobilized double enzyme, and free enzymes were observed by SEM. The CF surface was relatively smooth and neat (Figure 3a) [56]. After the treatment, some micron g-C_3_N_4_ was uniformly loaded on the surface of the g-C_3_N_4_/CF composite carrier (Figure 3b), resulting in the changed surface morphologies and rough surface of the carriers [29]. As shown in Figure 3c,d, the amount of immobilized double enzyme on the g-C_3_N_4_/CF surface was much more than that of on the CF; the dual enzyme was evenly distributed on the composite carrier in Figure 4d, which may be because the rough g-C_3_N_4_/CF surface provided more adsorption sites for the enzyme and enhanced the immobilized effect of the double enzyme.

The immobilized enzymes were tested by fluorescence microscopy to further analyze the distribution of lactase and glucose isomerase on the surface of the g-C_4_N_4_/CF composite carrier. As shown in Figure 4a, the surface of the composite carrier was obviously loaded with a lot of lactase and glucose isomerase. Figure 4b shows the red fluorescence of lactase labeled with Rhoda mine B. Figure 4c shows the green fluorescence of glucose isomerase labeled with FITC. Figure 4d shows that the two enzymes were uniformly distributed on the surface of the g-C_3_N_4_/CF composite carrier, which is beneficial for lactase to convert lactose into glucose and to then be utilized by glucose isomerase to fructose as soon as possible. The fructose and galactose are then used by nearby lactase to synthesize lactulose. The co-immobilization of lactase and glucose isomerase resulted in a multi-enzyme cascade reaction, which was beneficial to reduce the reactor volume, improved the production efficiency, and reduced waste generation [51].

**Figure 5:**
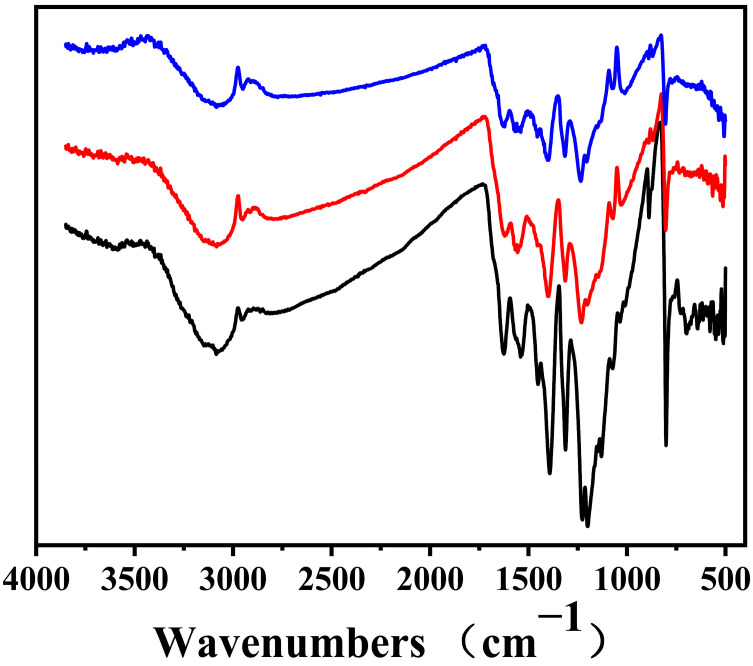
The FTIR spectra of g-C_3_N_4_, g-C_3_N_4_/CF, and g-C_3_N_4_/CF co-immobilized enzymes (g-C_3_N_4_/CF co-immobilized enzymes: blue line. g-C_3_N_4_/CF: red line. g-C_3_N_4_: black line).

The effect of the surface functional groups of the g-C_3_N_4_/CF composite carriers on dual-enzyme immobilization is shown in Figure 5. The surface functional groups of g-C_3_N_4_, g-C_3_N_4_/CF, and g-C_3_N_4_/CF co-immobilized enzymes were detected by FTIR.

The wide peak at 3500−3100 cm^−1^ was caused by the O–H stretching mode together with the vibrational mode of the N–H bond [28]. The characteristic peak at 1200−1700 cm^−1^ reflects the stretching vibration of the conjugated CN ring [29]. The characteristic peak at 804 cm^−1^ represents the respiratory vibration of the S-thiazine ring [57]. In addition, the characteristic peak of g-C_3_N_4_/CF was obviously weaker than that due to doping CF. Compared with g-C_3_N_4_/CF, the infrared spectrum intensity of g-C_3_N_4_/CF co-immobilized enzymes decreased significantly. The peaks of g-C_3_N_4_/CF co-immobilized enzymes at 1240, 1317, 1410, and 1625 cm^−1^ were attributed to aromatic C-N and C-N frameworks. These characteristic absorption peaks were highly overlapped with the absorption peaks of g-C_3_N_4_/CF, which was attributed to the physical interaction between the double enzyme and g-C_3_N_4_ [1]. The results showed that the basic surface structure of g-C_3_N_4_ was affected by the immobilization process.

In addition, some forces may exist between the enzyme and the surface of the composite carrier. The intensity of the amide band of the co-immobilized enzyme was reduced relative to the free double enzyme, from which it was inferred that the hydrophobic end of the enzyme interacts with the surface of the composite carrier. The immobilization process occurred due to hydrophobic forces, which contributed to the immobilization of the enzymes [58,59]. The N–H and H vibrational peaks of the g-C_3_N_4_/CF co-immobilized enzymes at 3500−3100 and 800 cm^−1^. It is the possible that the mechanism of immobilization was the bind of the hydrogen atoms on the enzyme surface to the amino groups of g-C_3_N_4_ on the surface of the g-C_3_N_4_/CF composite carrier under physical conditions [1].

### 3.3. Enzymatic Properties Analysis of the g-C_3_N_4_/CF Co-Immobilized Double Enzyme

The SDS-PAGE analysis of glucose isomerase and lactase were performed and results are displayed in Figure S2. Figure S2A,B both show only one band, indicating that all protein in both crude extracts corresponded to these enzymes. Thus, it was feasible for us to calculate the immobilization efficiency by determining the total protein content of the initial enzyme solution [60,61]. In a further, SDS profiles indicated that the molecular weight of lactase was about 120 kDa and that of glucose isomerase was about 70 kDa. The relative molecular mass of glucose isomerase reported in the relevant literature was about 63 kDa [62] and the molecular weight of lactase was between about 60 and 120 kDa [61,63], similar to the results we obtained.

The test conditions for immobilized enzymes had some effects on enzyme viability [64]. As shown in Table S2, lactase activity decreased from 5.90 U g^−1^ to 5.13 U g^−1^, with a decrease of 13%; glucose isomerase activity decreased from 58.94 U g^−1^ to 49.02 U g^−1^, with a decrease of 16.4%. The change in enzyme activity was small. The lactase and glucose isomerase with immobilization show the good stability of the experimental condition.

The immobilization process affected the distribution of the substrate at the enzyme active site [65]. The Michaelis constant Km is considered the most important constant for studying the kinetics of the enzyme reaction, which represents the affinity between the enzyme and the substrate. Larger Km values indicated weaker affinity and vice versa [46]. The Km of the co-immobilized enzymes was determined by using a series of concentrations of the lactose/fructose mixture (10% lactose and 2% fructose) as the substrate according to the Michaelis–Menten kinetics. As shown in Figure S3, the Km value of the co-immobilized enzyme was 0.09, indicating that the co-immobilized enzyme had a favorable affinity for the substrate.

**Figure 6:**
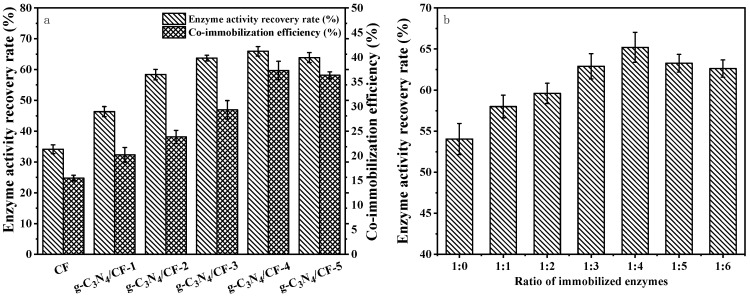
Effect of different carriers and enzyme activity ratios on co-immobilized enzyme activity. (**a**) Effect of different carriers on enzyme activity recovery and co-immobilization efficiency; (**b**) effects of double enzyme ratio on enzyme activity recovery of g-C_3_N_4_/CF co-immobilization enzyme.

The optimal preparation of g-C_3_N_4_/CF composite carrier was selected by enzyme activity recovery and co-immobilization efficiency. As shown in Figure 6a, 100% enzyme activity recovery rate referred to the enzyme activity of the free dual enzyme prior to immobilization at 55 °C, pH 6.0, which was 19.07 U g^−1^. During the preparation of the composite carriers, the recovery of enzyme activity and co-immobilization efficiency of the immobilized carriers gradually increased with the increase of melamine content. The enzyme activity recovery and co-immobilization efficiency of g-C_3_N_4_/CF-4 were 1.93 and 2.41 times that of CF, respectively. The g-C_3_N_4_/CF-4 had a higher co-immobilization efficiency of 37.31%. The enzyme activity recovery rate and co-immobilization efficiency of the composite carrier, prepared by continuously increasing the amount of melamine, did not obviously increase. This may be due to the fact that the g-C_3_N_4_ attached to the surface of CF had reached saturation in the modification process, leading to the properties of the composite carrier not being changed with the excessive melamine. Therefore, the g-C_3_N_4_/CF-4 was selected for subsequent experiments. For the convenience of description, the g-C_3_N_4_/CF composite carrier mentioned in the subsequent experiments only represents g-C_3_N_4_/CF-4.

The optimal ratio of immobilized lactase and glucose isomerase was determined by the enzyme activity recovery rate. As shown in Figure 6b, 100% enzyme activity recovery referred to the enzyme activity of the free double enzyme at each enzyme activity ratio, in which the enzyme activity of the free double enzyme was 19.5 U/g at the ratio of enzyme activity of 1:4. With the increase of the lactase and glucose isomerase activity ratio, the recovery rates of co-immobilized enzyme first increased and then reduced. When the glucose isomerase was insufficient, the glucose generated by lactose catalyzed by lactase inhibited the galactose conversion activity of lactase non-competitively, thus affecting the production of lactulose. This is similar to the reason for which Long et al. used glucose oxidase to relieve the inhibition of glucose to the fructose conversion activity of β-fructofuranosidase [3]. When the enzyme activity ratio was 1:4, the enzyme activity recovery rate was the highest, which may be related to the increase of glucose isomerase content promoting the conversion of glucose into fructose, thus promoting the increase of fructose concentration. In addition, the glucose conversion alleviated the inhibition of the galactose transfer activity of lactase, with the increased concentration of galactose. The increase of fructose and galactose promoted the production of lactulose. Subsequently, the enzyme activity recovery rate began to decrease, which may be due to the relative decrease of the active site binding lactase on the carrier surface with the excessive glucose isomerase. If glucose isomerase is excessive, the active sites of binding to lactase on the carrier surface would decrease relatively. Therefore, the enzyme activity ratio of lactase to glucose isomerase of 1:4 was selected for the subsequent experiments.

In the enzymatic catalytic process, the synthesis efficiency of the product depends not only on the preparation of the immobilized enzyme, but also on catalytic reaction conditions [66]. Temperature is one of the most important factors affecting enzyme catalysis.

**Figure 7:**
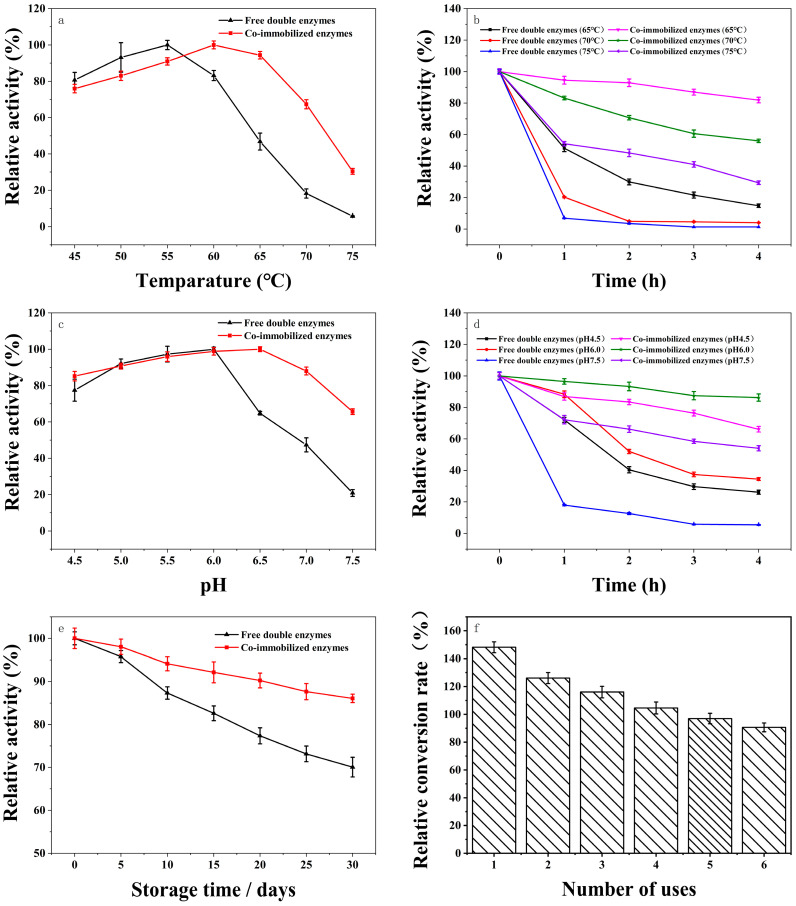
Enzymatic properties analysis of the g-C_3_N_4_/CF co-immobilized double enzyme. (**a**) The optimal reaction temperature changes of the free enzyme and the co-immobilized enzyme; (**b**) thermal stability of the free enzymes and the co-immobilized enzymes; (**c**) optimum pH change of the free enzyme and the co-immobilized enzyme; (**d**) the pH stability of the free enzymes and the co-immobilized enzymes; (**e**) storage stability of the free enzymes and co-immobilized enzymes; (**f**) lactulose relative conversion rate.

The optimal temperature of the free and co-immobilized enzymes was determined at 45–75 °C with a pH of 6.0. In Figure 7a, the relative activity of 100% referred to the enzyme activity at the optimum temperature. The activity of the free double enzyme was 19.19 U g^−1^ at 55 °C. The activity of the co-immobilized enzyme was 13.89 U g^−1^ at 60 °C. As shown in Figure 7a, with the increase in temperature, the relative activities of immobilized and free double enzymes showed a trend of increasing and then decreasing. The decrease or loss of enzyme activity at a high temperature was due to the structural fragility of the enzyme and enzymatic dissociation [67]. The optimum temperature of the co-immobilized enzyme increased by 5 °C, indicating that the co-immobilized enzyme had higher thermal stability than the free enzyme. At the same time, the activity of the co-immobilized enzyme was significantly higher than that of the free enzyme at the temperature of 60–75 °C, indicating that the co-immobilized enzyme had the wider range of temperature application.

In order to further investigate the effect of immobilization on the thermal stability of the enzyme, the enzyme activities of free enzymes and co-immobilized enzymes were measured at 65 °C, 70 °C, and 75 °C with the reaction time. In Figure 7b, the relative activity of 100% referred to the initial enzyme activity with different temperatures at pH 6.0. The activity of the free double enzyme was 18.77 U g^−1^ and the activity of the co-immobilized double enzyme was 13.80 U g^−1^. As shown in Figure 7b, the inactivation rate of free enzymes at the same high temperature over time was much higher than that of the co-immobilized enzyme, indicating that the co-immobilized enzyme had higher thermal stability, which was attributed to the increased rigidity of enzyme molecules with immobilization, preventing enzymatic subunit dissociation due to thermal denouement [68]. The chemical bonds formed during physical bonding could protect enzyme molecules from conformational changes at high temperatures [1].

pH is also one of the most important factors affecting enzyme activity, because the H^+^ concentration in the environment not only affects the quaternary structure of the enzyme, but also affects the degree of ionization of the substrate, product, and active site residue [67]. With the increase of pH, the relative activities of co-immobilized and free double enzymes showed a trend of increasing and then decreasing. The free double enzymes and co-immobilized enzymes had the maximum relative activities at pH 6.0 and pH 6.5, respectively.

The changes in the activities of free and co-immobilized enzymes may be due to the effect of pH on the structural stability of the enzyme molecule, where very high or low pH levels denatured the protein deactivating the enzyme. Secondly, the change of pH value may also affect the dissociation state of enzymes and substrates [3]. The optimum pH values of free enzymes and co-immobilized enzymes were determined at 55 °C and a pH of 4.5–7.5.

In Figure 7c, the relative activity of 100% referred to the enzyme activity of at the optimum pH at 55 °C. The activity of the free double enzyme was 19.55 U g^−1^ at pH 6.0. The activity of the co-immobilized enzyme was 13.75 U g^−1^ at pH 6.5. As shown in Figure 7c, the optimal pH values of the free dual enzymes and the co-immobilized enzymes were 6.0 and 6.5, respectively. When the pH value was higher than 6.5, the wider pH stability was displayed in the co-immobilized enzymes, rather than the free dual enzymes. When the pH value was 7.5, the relative activity of the co-immobilized enzymes remained at 65.65% of the original enzyme activity, while the relative activity of the free enzymes decreased to 20.88%. The shift of optimal pH to the alkaline range may be related to the conformational changes of the enzyme on the carrier along with the protonation of the microenvironment. Electrostatic interactions between the pH and surface of the enzyme molecule leads to changes in the enzyme active center and conformational changes of the enzyme [24].

To further investigate the effect of immobilization on the pH stability of the enzyme, the activities of free enzymes and co-immobilized enzymes were measured after the incubation at pH of 4.5, 6.0, and 7.5 for different times. In Figure 7d, the relative activity of 100% referred to the initial enzyme activity with different pH values at 55 °C. The activity of the free double enzyme was 18.64 U g^−1^. The activity of the co-immobilized double enzyme was 13.13 U g^−1^. As shown in Figure 7d, the co-immobilized enzymes had a lower inactivation rate and wider pH stability compared to the free enzymes, which may be due to the fact that immobilized carriers could act as ion exchangers, making the pH of the solution around the enzyme different from that of the substrate solution [3].

It is known that enzyme leakage is an important factor on the effect of enzyme immobilization technology [66]. Thus, the stability of co-immobilized enzymes during operation and storage was investigated, which was the key performance index for practical applications.

To further explain the decrease in enzyme reusability, the storage stabilities of the enzyme were tested. In Figure 7e, the relative activity of 100% referred to the initial enzyme activity for storage. The activity of the free double enzyme was 19.29 U g^−1^. The activity of the co-immobilized double enzyme was 13.57 U g^−1^. As shown in Figure 7e, after 30 days of storage at 4 °C, the activity of co-immobilized enzyme was retained at 86.08%, while the activity of the free enzyme was retained at 70.06%, indicating that the storage stability of co-immobilized enzymes was significantly higher than that of free enzymes. The operating stability of the free double enzyme and co-immobilized enzymes were measured at 65 °C and a pH of 6.0, as shown in Figure 7f, where the free enzyme was not easily recovered and reused. In Figure 7f, the relative conversion of 100% was the amount of lactulose produced by the breakdown of the substrate with the free enzyme at 65 °C. The concentration of lactulose at this point was 7.19 g L^−1^.

The relative conversion rate of the co-immobilized enzyme was higher in the first four batches than that of the free enzyme, remaining at around a 90% relative conversion rate after six cycles. The main reason why the co-immobilized enzymes were able to achieve multi-batch production was the positive effects of the composite carrier on the enzyme molecules. It was shown that the composite carrier with the large specific surface area and appropriate pore sizes enhanced the immobilization efficiency of enzymes [69]. The composite carrier had a rough surface with more enzyme molecules’ immobilization than that of the CF, which may be due to the fact that the rough surface of the composite carrier could provide the larger binding area to increase the enzymes’ immobilization efficiency. Furthermore, the images from laser scanning confocal microscopy indicated that the double enzymes were uniformly distributed on the composite carrier, which improved the immobilized enzymes for the production of lactose from the substrate. The presence of these conditions altered the microenvironment surrounding the enzyme molecule, which showed that the conformation of immobilized enzyme did not change much [68]. The negative effects outside on the co-immobilized enzymes were reduced by the co-immobilization. Furthermore, the enzymes were distributed on the surface of the carrier, which reduced the accumulation of enzymes during the storage of free enzymes [66]. Consequently, the stability of co-immobilized enzymes was raised, to the benefit of lactulose production, in multiple batches.

## 4. Conclusions

In this paper, g-C_3_N_4_/CF composite carriers were prepared by ultrasonically assisted impregnation and high-temperature calcination for the co-immobilization of lactase and glucose isomerase. The results showed that the surface of g-C_3_N_4_/CF composite carriers were uniformly composited with some certain microns of g-C_3_N_4_, which made the surface rougher, increased the specific surface area, optimized the pore structure, and provided more adsorption sites for the enzymes. The immobilization efficiency of 37% and enzyme activity of 13.89 U g^−1^ were obtained in the co-immobilized enzyme by the carrier of g-C_3_N_4_/CF. The lactase and glucose isomerase co-immobilized with the g-C_3_N_4_/CF composite carrier displayed good stability. Moreover, compared to the free enzymes, the co-immobilized enzymes performed higher relative conversion rates of lactulose during the multi-batch production. Therefore, the g-C_3_N_4_/CF composite carrier has wide application prospects in the immobilization of lactase and glucose isomerase for lactose production.

## Figures and Tables

**Table 1:** Specific surface area, pore volume, and pore size of different carriers.

	CF	g-C_3_N_4_/CF
S_BET_ (m^2^ g^−1^)	0.61 ± 0.04	5.08 ± 0.04
Microporous volume (cm^3^ g^−1^)	1.91 × 10^−4^ ± 1.14 × 10^−5^	2.15 × 10^−3^ ± 1.39 × 10^−5^
Mesoporous volume (cm^3^ g^−1^)	1.76 × 10^−3^ ± 2.24 × 10^−4^	2.75 × 10^−2^ ± 1.23 × 10^−3^
Total pore volume (cm^3^ g^−1^)	1.95 × 10^−3^ ± 2.27 × 10^−4^	2.96 × 10^−2^ ± 1.23 × 10^−3^
Average pore diameter (nm)	12.87 ± 1.65	23.35 ± 0.97

## Data Availability

Not applicable.

## Supplementary Materials

The following supporting information can be downloaded at: https://www.mdpi.com/article/10.3390/nano12234290/s1, Table S1: Surface element composition of CF, g-C_3_N_4_, and g-C_3_N_4_/CF.; Figure S1: N_2_ adsorption-desorption isotherms and pore size distributions of different carriers. (a) N_2_ adsorption-desorption isotherms for CF and g-C_3_N_4_/CF (Adsorption isotherm plot of CF, black line. Desorption isotherm plot of CF, red line. Adsorption isotherm plot of g-C_3_N_4_/CF, blue line. Desorption isotherm plot of g-C_3_N_4_/CF, purple line). (b) Pore size distribution of CF and g-C_3_N_4_/CF (The CF, black line. The g-C_3_N_4_/CF, red line).; Figure S2: SDS-PAGE analysis of glucose isomerase and lactase. In each sample lane, 10.0 µL of protein sample was added. Lane M: protein marker, lane A: glucose isomerase, lane B: lactase.; Table S2: Effect of immobilization conditions on enzyme activity.; Figure S3: Lineweaver–Burk plots of the co-immobilized enzyme. The Km of the immobilized and free enzymes was determined by using a series of concentrations of the lactose\fructose mixture (10% lactose and 2% fructose) as the substrate according to the Michaelis-Menten kinetics.
