# Supplementary material for: Co-Immobilization of Lactase and Glucose Isomerase on the Novel g-C3N4/CF Composite Carrier for Lactulose Production
Source: Nanomaterials (Basel). 2022 Dec 2;12(23):4290. doi: 10.3390/nano12234290 (PMC9738431; doi:10.3390/nano12234290)
Supplement: Supplementary file 1 [file nanomaterials-12-04290-s001.zip › nanomaterials-2032773-SI.pdf]

Supplementary materials

Table S1. Surface element composition of CF, g-C_3N_4, and g-C_3N_4/CF

Carrier type	Element content (%)				
	C	O	N	O/C	N/C
CF	81.22	17.54	1.24	21.59	1.53
g-C_3N_4	44.79	2.69	52.52	6.01	117.3
g-C_3N_4/CF	72.44	13.52	14.03	18.66	19.37

Note: The CF referred to the carbon fibers without reacting with melamine. The g-C_3N_4 referred to the pure g-C_3N_4, which was synthesized by calcining the melamine directly in 550℃ for 3 h. The g-C_3N_4/CF referred to the g-C_3N_4/CF composite carrier synthesized by being calcined with melamine and CF in 550℃ for 3 h.

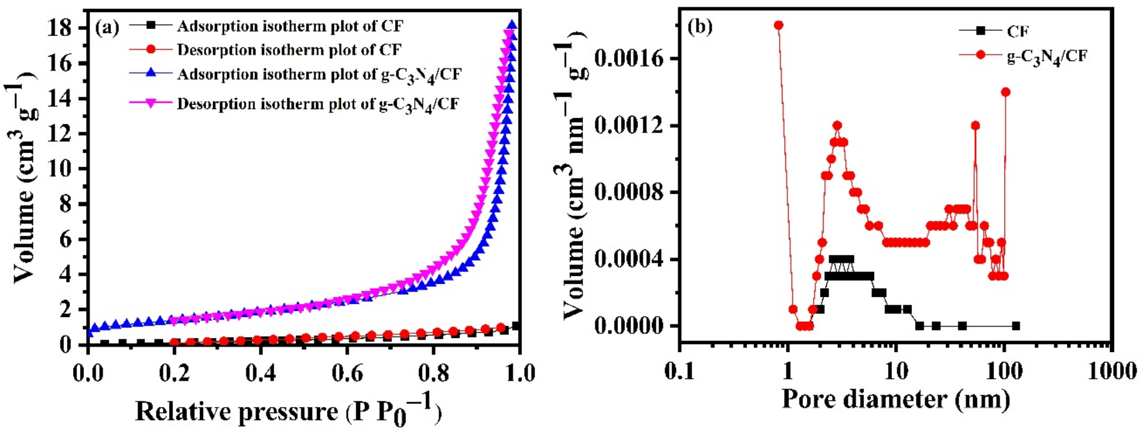

Figure S1. N_2 adsorption-desorption isotherms and pore size distributions of different carriers. (a) N_2 adsorption-desorption isotherms for CF and g-C_3N_4/CF (Adsorption isotherm plot of CF, black line. Desorption isotherm plot of CF, red line. Adsorption isotherm plot of g-C_3N_4/CF, blue line. Desorption isotherm plot of g-C_3N_4/CF, purple line.). (b) Pore size distribution of CF and g-C_3N_4/CF (The CF, black line. The g-C_3N_4/CF, red line).

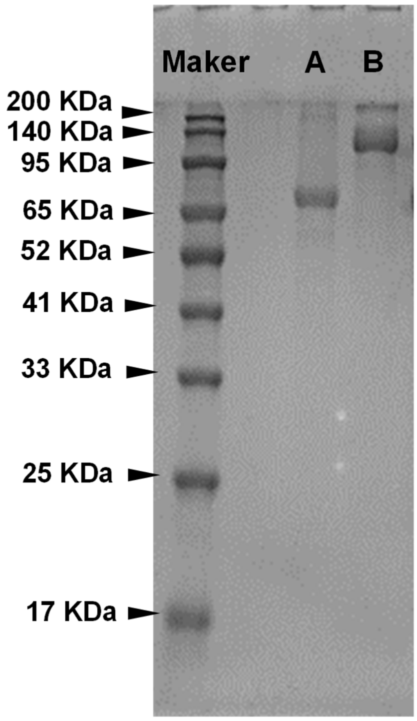

Figure S2. SDS-PAGE analysis of glucose isomerase and lactase. In each sample lane, 10.0 µl of protein sample was added. Lane M: protein marker, lane A: glucose isomerase, lane B: lactase.

Table S2. Effect of immobilization conditions on enzyme activity

	Lactase activity ($U\ g^{-1}$)	Glucose isomerase activity ($U\ g^{-1}$)
Before the immobilization conditions	5.90±0.07	58.94±1.60
After the immobilization conditions	5.13±0.09	49.02±0.35

Notes: the immobilization condition referred to the shaking at 30°C, 160 r min^{-1}, for 5 h.

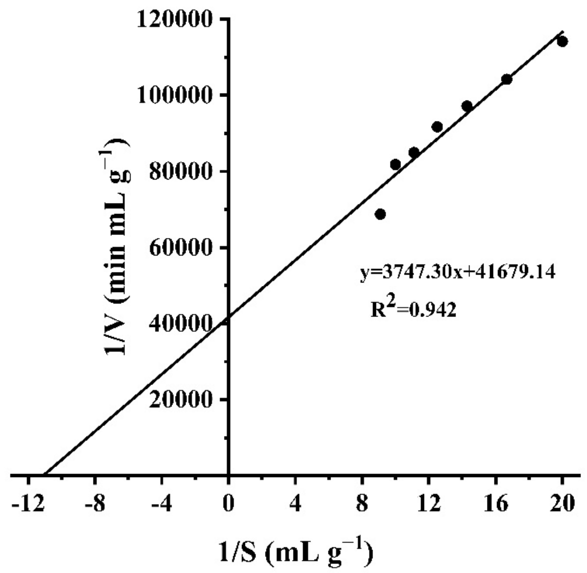

Figure S3. Lineweaver-Burk plots of the co-immobilized enzyme. The Km of the immobilized and free enzymes was determined by using a series of concentrations of the lactose/fructose mixture (10% lactose and 2% fructose) as the substrate according to the Michaelis-Menten kinetics.
